# A phase II trial of the effect of perindopril on hand–foot skin reaction (HFSR) incidence and severity in patients receiving regorafenib for refractory mCRC

**DOI:** 10.1007/s00280-018-3738-x

**Published:** 2018-12-08

**Authors:** Barbara L. Melosky, Howard John Lim, Janine Marie Davies, Sharlene Gill, Christian K. Kollmannsberger, Maria Yi Ho, Solomon A. Vandt, Daniel John Renouf

**Affiliations:** BC Cancer Vancouver Centre, 600-10th Ave W, Vancouver, BC V5Z 4E6 Canada

**Keywords:** Regorafenib, Colorectal cancer, Perindopril, ACE inhibitor, HFSR

## Abstract

**Purpose:**

Regorafenib is an oral multi-kinase inhibitor that offers an OS benefit to patients with mCRC refractory to standard therapy (Grothey et al., in Lancet 381:303–312, 2013), but comes with potential significant toxicities including grade 3 hand–foot skin reaction (HFSR). The pathogenesis of regorafenib-induced HFSR is not well established, but may be related to alterations in the capillary endothelium. We hypothesized that perindopril, an angiotensin-converting enzyme (ACE) inhibitor, indicated for the treatment of hypertension (Ceconi et al., in Cardiovasc Res 73:237–246, 2007), and which plays a role in preventing endothelial dysfunction, may help to prevent or reduce the severity of regorafenib-induced HFSR.

**Patients and methods:**

In this single-center phase II open-label trial, patients with refractory mCRC were treated with both regorafenib (160 mg/day) and perindopril (4 mg/day) for 21 days per 28-day cycle. The primary end point was to assess the proportion of patients with any grade HFSR toxicity. Secondary end points included time to development of worst (grade 3) HFSR, reduction of all grades of hypertension and all grade toxicities, as well as progression-free survival. All toxicities were evaluated using CTCAE v4.03.

**Results:**

A planned interim analysis was performed after ten evaluable patients had completed their first cycle of study treatment. As 50% (5/10) experienced grade 3 HFSR, enrolment was stopped as the addition of perindopril did not lead to a reduced level of HFSR compared with regorafenib alone. Other grade 3 toxicities included hypertension (16.7%) and increased AST (16.7%).

**Conclusion:**

The addition of an ACE inhibitor perindopril to regorafenib did not reduce HFSR incidence or severity in patients with refractory mCRC.

## Purpose

Colorectal cancer is the second most frequently diagnosed cancer in males and the third most frequently diagnosed cancer in females [1]. The disease remains the second most frequent cause of cancer death in males and the third most frequent cause of cancer death in females [1].

The median overall survival (OS) for patients with unresectable mCRC who receive only best supportive care (BSC) is 5–6 months [2]. Palliative treatment with systemic chemotherapy prolongs survival and maintains quality of life [3].

For many years, 5-fluorouracil (5-FU) was the only treatment, but the approval of irinotecan, oxaliplatin, fluoropyrimidines, as well as monoclonal antibodies such as bevacizumab targeting VEGF and cetuximab and panitumumab targeting EGFR growth factors, led to the development of additional regimens. The ideal combination and sequence of the different agents are still being determined.

Regorafenib has shown efficacy in mCRC patients who have been previously treated with fluoropyrimidine-based chemotherapy, oxaliplatin, irinotecan, an anti-VEGF therapy, and, if *RAS* wild type, an anti-EGFR therapy [4, 5]. It is a multi-kinase inhibitor belonging to a unique class of orally administered small molecule therapeutics targeting multiple protein kinases, including kinases involved in tumour angiogenesis (VEGFR1, -2, -3, TIE2), oncogenesis (KIT, RET, RAF-1, BRAF, BRAFV600E), and the tumour microenvironment (PDGFR, FGFR) [6]. The phase III CORRECT trial demonstrated regorafenib’s efficacy in mCRC patients who were refractory to standard therapy, in which regorafenib prolonged OS compared with placebo [4]. The efficacy and safety results were confirmed in the randomised CONCUR trial [5]. The study met its primary OS end point, 8.8 months for regorafenib as compared to 6.3 months with placebo [HR 0.550, 95% CI (0.395–0.765), *P* = 0.0002 (1-sided)].

Hand–foot skin reaction (HFSR) is the most significant AE experienced by regorafenib-treated patients. Efforts are needed to reduce HFSR related to regorafenib, as it causes a significant decrease in a patient’s quality of life, requires dose reductions or modifications, and can ultimately lead to treatment discontinuation. In the CORRECT and CONCUR trials, 47–74% of patients treated with regorafenib experienced any grade of HFSR, with about 17% experiencing grade 3 HFSR [4, 5]. Skin toxicity was also the most frequent adverse event necessitating dose modification or interruption.

The pathogenesis of regorafenib-induced HFSR has not yet been fully established; however, the capillary endothelium may be the first target in HFSR. Abnormal signalling interrupting the VEGF and PDGF receptors may lead to alteration of small vessels [7], which are traumatized by frequent impacts or pressure requiring endothelial repair [8]. An inflammatory response has also been hypothesized [8].

Hypertension is also an issue with regorafenib, affecting up to 28% of patients in the CONCUR and CORRECT trials, with 12 and 7% reporting severe hypertension, respectively [4, 5].

Perindopril is an angiotensin-converting enzyme (ACE) inhibitor indicated for the treatment of hypertension, congestive heart failure, and for the treatment of hypertensive and/or post-MI patients with stable coronary artery disease [9]. ACE inhibitors protect the vascular endothelium by decreasing the concentration of angiotensin II, which inhibits nitrous oxide (NO) production and activity while increasing the concentrations of bradykinin, a vasodilator and stimulator of NO [10]. Different ACE inhibitors have demonstrated differing abilities to improve vascular endothelial function as demonstrated through flow-mediated vasodilation [11], with perindopril being most effective [10]. As a vasodilator, perindopril may be beneficial in preventing or reducing the severity of regorafenib-induced HFSR, possibly by helping to restore the normal balance between angiotensin II and bradykinin, and reducing inflammation (TNF-a) to reverse endothelial dysfunction [10, 12–14].

The hypothesis underlying this trial was that the co-administration of perindopril with regorafenib would mitigate HFSR symptoms, increasing the treatment duration.

## Methods

### Study design and participants

This phase II Study of Perindopril and Regorafenib in Metastatic Colorectal Cancer (PARICCA) was an open label, single arm trial of patients with refractory mCRC, conducted to measure the incidence and severity of HFSR and hypertension using the CTCAE v4.03 criteria, in patients receiving both regorafenib (160 mg/day) and perindopril (4 mg/day).

Eligible patients had a pathologic documentation of stage IV adenocarcinoma of the colon or rectum and progressed on/after all approved drugs for mCRC including FU-based chemotherapy, oxaliplatin, irinotecan, anti-VEGF therapy, and, if *RAS* wild type, an anti-EGFR therapy. Patients had to have refractory or progressive disease within 3 months following the last administration of approved standard therapies, or experienced intolerance to previous therapy. Patients treated with oxaliplatin in the adjuvant setting had to progress during or within 6 months of completion of adjuvant therapy. Patients were aged 18 years or older; had an Eastern Cooperative Oncology Group (ECOG) performance status of 0 or 1 within 14 days prior to the initiation of study treatment; life expectancy of at least 3 months; and adequate bone marrow, liver and renal function at the start of the trial. Patients were not eligible if they had previously received regorafenib; had an uncontrolled medical disorder deemed significant by the treating physician; and history of hereditary/idiopathic angioedema, or angioedema related to previous treatment with an ACE inhibitor.

The study was conducted at the BC Cancer Vancouver Center. The University of British Columbia Research Ethics Board approved the protocol, ClinicalTrials.gov Identifier NCT02651415. The trial followed the guiding principles of the Declaration of Helsinki and good clinical practice and complied with all local laws and regulations. Participants provided written informed consent before enrolment; when a patient was not capable of providing a signature, an oral statement of consent could be provided in the presence of a witness.

### Study end points

The primary end point was the incidence of all grade toxicities for HFSR defined by CTCAE v4.03 criteria. The incidence of HFSR was expressed as the number of patients experiencing any grade HFSR. The primary end point was a 50% reduction in all grades of HFSR based on CTCAE v4.03 criteria (i.e. from 47% any grade HFSR to 24% of patients).

Secondary end points were the incidence of all grade hypertension and toxicity; maximal severity of HFSR; time to development of stage 3 HFSR; and progression-free survival (PFS). All grades of hypertension were evaluated using CTCAE v4.03, measured weekly for the first 6 weeks while patients were on the study drug, then every second week during treatment with perindopril and during the 30-day follow-up period (post-therapy).

All grades of AE (including HFSR) were evaluated using CTCAE v4.03 at baseline and day 1 of each cycle while they were on the study drug and during the 30-day follow-up period (post-therapy). PFS was evaluated based on RECIST v1.1 criterion, with 20% progression of existing metastases or any new metastases.

Information on previous experience with HFS was collected as per patient recollection only at enrolment (no chart review). This was an exploratory analysis to determine if previous HFS influences the rate and severity of HFSR in this study.

Patients were followed for survival. For subjects who discontinued study treatment and did not experience PD, available tumour assessments were recorded in the CRF until documented PD.

### Procedures

The dose and schedule of regorafenib used in this trial was the approved dose of 160 mg per day from data accumulated in previous regorafenib phase III trials [4, 5]. Perindopril erbumine 4 mg was administered daily for 21 days of a 28–day cycle [9]. Perindopril was administered in the morning on an empty stomach. Regorafenib was administered 160 mg daily for 21 days of a 28–day cycle, with a low fat breakfast 1 h after perindopril. All patients received BSC.

Predefined dose modifications were permitted to manage clinically significant treatment-related AEs. Patients who required dose reductions could re-escalate the dose up to 160 mg daily at the discretion of the investigator once the AE resolved to baseline levels. Treatment was discontinued permanently if the AE did not recover after a 4-week interruption or after dose reduction by two dose levels.

Patients were treated until clinical radiological disease progression based on RECIST v1.1 criterion, death, unacceptable toxicity, withdrawal of consent by the patient, decision by the treating physician that discontinuation would be in the patient’s best interest, or substantial non-compliance with the protocol. If in the investigator’s opinion treatment with regorafenib provided clinical benefit to a patient experiencing disease progression, the patient could continue treatment.

Patients were seen by a physician every week for the first cycle, every 2 weeks for the second cycle, at the start of each subsequent cycle, at the end of treatment, and every month after cessation of treatment until death or data cutoff. ECOG status and concomitant medications were assessed at the start of treatment and at each physician visit. Blood pressure and LFTs were done weekly for the first cycle, on days 1 and 7 in the second cycle, on day 1 of each subsequent cycle, and at the follow-up visit. Lipase, electrolytes, TSH, and chemistry were collected on day 1 of each cycle. CEA, CBC, and creatinine were evaluated on day 1 of each cycle and at the end of treatment. A 12-lead ECG was administered on day 11 of each cycle and at the end of treatment. Other tests were administered as per the standard of care for regorafenib and perindopril. The study protocol mandated a patient education module that included a patient education brochure regarding the management of HFSR and preventative measures to manage this side effect.

### Study interim analysis and discontinuation rule

The study was to be discontinued if five of the first ten patients exhibited a Grade 3 HFSR, or if the HFSR was more severe with the addition of perindopril than with regorafenib alone.

### Statistical analysis

Demographic and other baseline characteristics were listed and summarized. Qualitative data were summarized using frequencies and percentages; quantitative data were summarized using descriptive statistics. Secondary safety variables were summarized using descriptive statistics and exploratory graphical presentations of the data. Statistical analyses were performed using SAS 9.3.

The primary analysis set consisted of all evaluable patients. An evaluable patient was defined as an eligible patient who received at least one cycle of study medication. The safety set consisted of all patients who received at least one dose of study medication. RECIST criteria were used by the investigator to determine PFS and OS end points.

### Sample size assumptions

A 10% reduction in all grades of HFSR is a clinically meaningful reduction, with an alpha level of 0.05 and a power of 80% for the sample size calculation.

## Results

This study was initiated in August 2016 and the last patient visit was conducted in March 2018. Although 12 patients were accrued over a 9-month period, 2 patients withdrew in the first cycle; as such, 10 patients were evaluable.

The baseline characteristics of the patients are shown in Table 1. Equal numbers of males and females (5:5) were enrolled, and the median age of the trial participants was 60 years (range 49–72), and at the baseline screening assessment 70% had ECOG 1 performance status. Forty percent of patients had experienced previous HFS, most likely from previous therapy.

**Table 1:** Baseline demographics in the evaluable patient population

	*N* = 10 (%)
Age (years)	Median 60.65 (range, 49– 72)
Sex	
Female	5 (50%)
Male	5 (50%)
Baseline ECOG	
0	3 (30%)
1	7 (70%)
Ethnicity	
Asian	5 (50%)
Black	1 (10%)
White	4 (40%)
Previous HFSR or HFS	
Yes	4 (40%)
If yes, and had HFSR during the study	3 (30%)
Stage at diagnosis	
II	1 (10%)
III	4 (40%)
IV	5 (50%)
Previous systemic anticancer therapies	
2	3 (30%)
3	6 (60%)
4	1 (10%)

Seven patients had moderately differentiated tumours, seven had three or more previous systemic anticancer therapies for metastatic disease, and eight patients had received FOLFOX or FOLFIRI before starting on this therapy.

The median days on treatment was 51.9 days. Four patients completed three or four treatment cycles (Table 2). Half of the patients received a dose reduction in this study, with three requiring one dose reduction and two requiring two dose reductions. Nine patients had a dose delay or hold, with five experiencing a delay of ≥ 1 week and 40% experiencing a delay of ≥ 2 weeks. Seven discontinued treatment due to disease progression.

**Table 2:** Treatment: dose levels

Mean days of treatment (*N* = 10)	55.9 (6–118) days
Total number of cycles received	*N* = 10 (%) (evaluable)
1	2 (20%)
2	4 (40%)
3	2 (20%)
4	2 (20%)
Lowest dose reduction level	
0 (160 mg PO OD)	5 (50%)
− 1(120 mg PO OD)	3 (30%)
− 2 (80 mg PO OD)	2 (20%)
Longest dose delay/hold	
No delays	1 (10%)
< 1 week	0 (0%)
≥ 1 week	5 (50%)
≥ 2 weeks	4 (40%)

After ten evaluable patients had completed their first cycle of study treatment, a planned interim analysis was conducted. As five of the patients enrolled experienced grade 3 HFSR, the study was discontinued, as the addition of perindopril was unlikely to lead to a reduced level of regorafenib-induced HFSR compared with regorafenib alone.

The most frequent grade 3 AEs were HFSR (50%), hypertension (20%), and increased AST (20%). All other toxicities are described in Table 3. Two patients experienced grade 1 or 2 HFSR. Interestingly, three of the four patients who had previous HFS experienced grade 3 HFSR while on this study.

**Table 3:** Clinical adverse events and laboratory measurements of all grades and of grade 3 and above

Any event	*N* all grades	*N* grade ≥ 3
*Clinical adverse event*		
Hand–foot skin reaction	7	5
Fatigue	7	2
Hypertension	6	2
Pain, abdomen	6	1
Anorexia	6	
Oral mucositis (dry mouth, sore throat, mucosal infection)	6	
Diarrhoea	5	
Muscle pain (myalgia)	5	
Rash or desquamation	4	1
Nausea	4	
Constipation	4	
Voice changes/hoarse voice	4	
Arthralgia (joint pain)	4	
Pain extremity/neuralgia	3	1
Infection (UTI)	3	
Fever (pyrexia)	3	
Dry skin	3	
Vomiting	3	
Ascites	3	
Headache	3	
Thigh, groin, pelvic pain	2	1
Non-cardiac chest pain	2	1
Back pain	2	
Rectal/anal pain	2	
Dysphagia (difficulty swallowing)	2	
Oedema (ankle)	2	
Dyspnoea (shortness of breath)	2	
Dry cough	2	
Pneumonia	1	1
Cramps (leg)	1	
Erythaema face	1	
Haemoptysis (spitting blood)	1	
Insomnia	1	
Pruritis (itchy)	1	
Pain, soles of feet, toe	1	
Weakness	1	
Chills	1	
Nose bleed (epistaxis)	1	
Weight loss	1	
Paraesthesia	1	
*Laboratory values*		
Increased AST (aspartate aminotransferase)	3	2
Hypokalaemia	2	1
Hyperbilirubinaemia	2	1
Increased alk phosphatase	1	1
Increased lipase	1	1
Elevated INR	1	
Haematuria	1	

The median days to HFSR resolution by one grade was 13 days (range 2–109 days) for all transitions (*n* = 17), 5.5 days (range 2–25) for grade 2 to grade 1 (*n* = 6), and 25 days (range 15–109 days) for grade 1–0 (*n* = 6). The number of days to complete HFSR resolutions from grade 3 to 0 was 51 days (range 36–125 days) (*n* = 5).

**Fig. 1:**
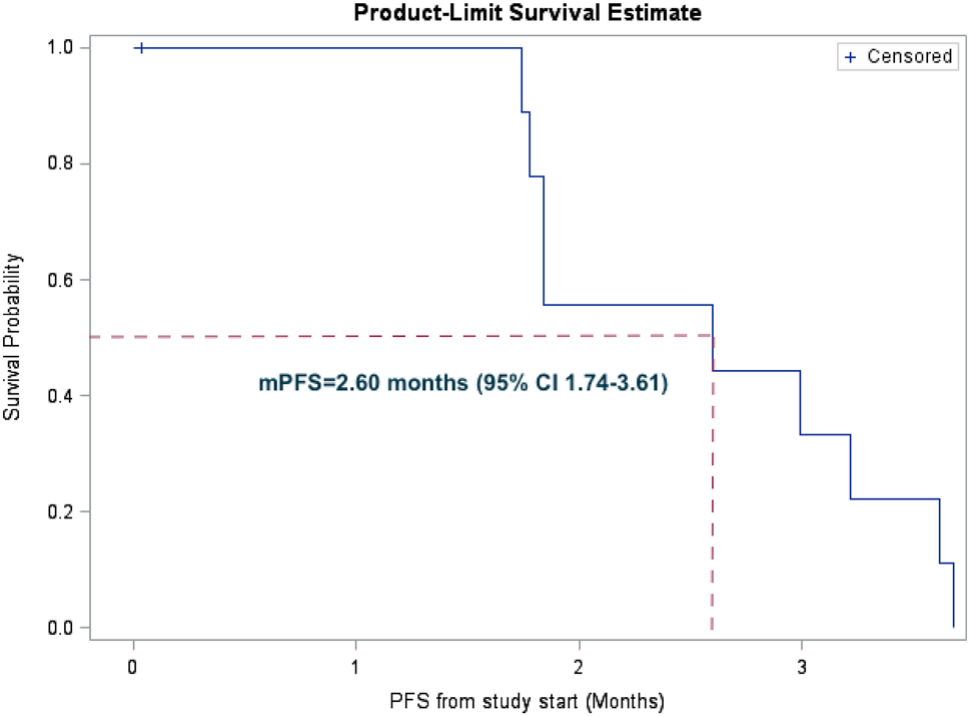
Kaplan–Meier curve of progression-free survival. One patient was censored and assumed to progress 1 day after start of study (as per protocol). This patient is indicated by ‘+’ at the start of the line on the plot

Patient outcomes are shown in Table 4. PFS from study start was 2.60 months (95% CI 1.74–3.61). The median OS was 7.33 months (95% CI 2.33–11.76). PFS and OS are shown in Fig. 1a, b, respectively.

**Table 4:** Outcomes for evaluable patients (*n* = 10)

Outcome	Months (95% CI)
Median PFS from study start	2.60 (1.74–3.61)
Median OS from study start (months)	7.33 (2.33–11.76)
Median OS from diagnosis of metastasis (months)	30.49 (12.19–43.53)

## Discussion

**Fig. 2:**
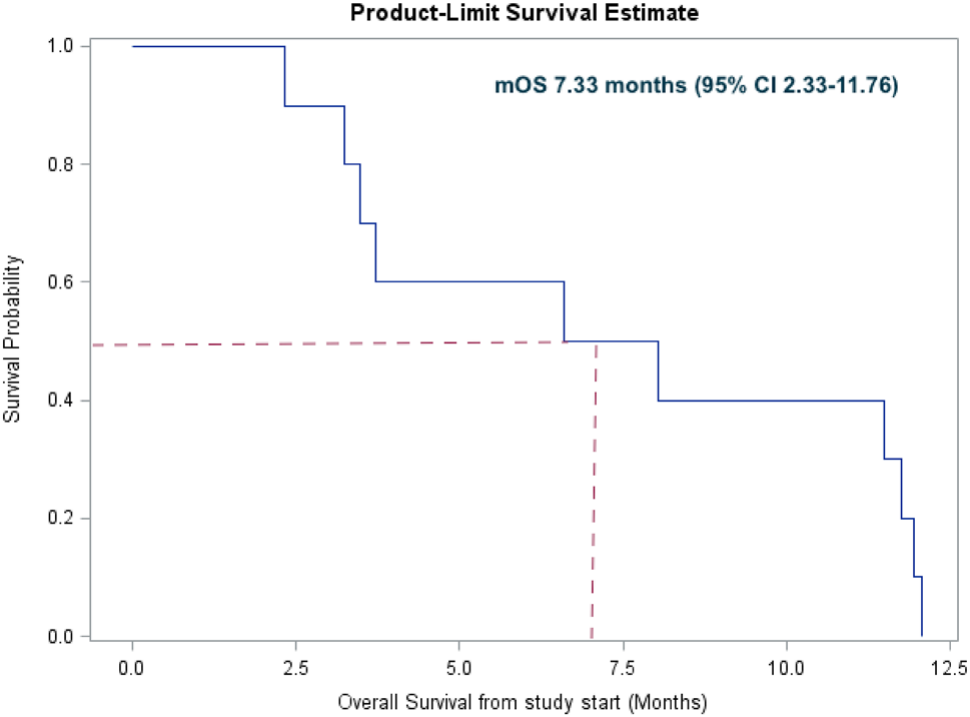
Kaplan–Meier curve of overall survival

Regorafenib has demonstrated OS benefits for patients with mCRC refractory to standard therapies. Regorafenib-induced HFSR can significantly impact the quality of life, requiring dose reductions or modifications. Our study objective was to determine if perindopril had any effect on the incidence of regorafenib-induced HFSR in refractory mCRC. Our hypothesis was that the vasodilation of small vessels with perindopril would mitigate regorafenib-associated HFSR and hypertension in an mCRC context. Although there were many different ACE inhibitors to consider, we chose perindopril as it is least likely to cause hypotension. The results demonstrated that the addition of perindopril to regorafenib did not reduce the rates of HFSR, and the trial was discontinued at the interim analysis due to high rates of grade 3 HFSR (Fig. 2).

Although the study numbers were small, the rate of grade 3 HFSR was much higher than expected based on a previous trial. In the CORRECT and CONCUR trials, only 17% and 16.2% of patients experienced grade 3 HFSR [4, 5].

An interesting observation from this small study was that patients with a previous (self-reported) history of HFS secondary to capecitabine had a high rate of regorafenib-induced HFSR. The duration of time to resolution of HFSR was long in this trial, especially for grade 3 HFSR to resolve completely (51 days). Previous HFS and duration of time to resolution may be considered in future regorafenib studies.

Efforts need to be made to find new dosing strategies and therapeutic combinations to reduce HFSR. One promising strategy is the Regorafenib Dose Optimization Study (ReDOS) [15]. This randomized phase II study examined initiating regorafenib at low doses (80 mg) and then escalating to 160 mg, with the end point of increasing the proportion of patients who completed two cycles of treatment and initiated a third, compared to patients who started on the standard dose. The initial results indicated that the study met its primary end point with 43% of the patients in the dose escalation cohort initiating a third dose versus 25% of patients on standard therapy. Also, median OS, PFS and overall rates of grade 3/4 toxicity were more favourable in the dose escalation arm. This study also tested the use of a steroid cream, clobetasol propionate, to reduce HFSR, but the results for this are yet to be reported.

Further studies to reduce HFSR for regorafenib and other multi-targeted tyrosine kinase inhibitors are needed, as these may significantly impair the optimal treatment benefit and the patient’s quality of life.

## Abbreviations

AE: Adverse event
ALT: Alanine aminotransferase
AST: Aspartate aminotransferase
BCCA: BC Cancer Agency
BSC: Best supportive care
CBC: Complete blood count
CRC: Colorectal cancer
CTC(AE): Common Terminology Criteria (for AEs)
ECOG: Eastern Cooperative Oncology Group
EGFR: Epidermal growth factor receptor
FGFR: Fibroblast growth factor receptor
FU: 5-Fluorouracil
HR: Hazard ratio
HFSR: Hand–foot skin reaction
KM: Kaplan–Meier
mCRC: Metastatic colorectal cancer
OS: Overall survival
PARRICA: Perindopril and regorafenib in mCRC
PDGF: Platelet-derived growth factor
PDGFR: Platelet-derived growth factor receptor
PFS: Progression-free survival
PS: Performance status
RECIST: Response Evaluation Criteria in Solid Tumors
VEGF: Vascular endothelial growth factor
VEGFR: Vascular endothelial growth factor receptor
vs: Versus
